# Strong light-matter coupling and field quantization in a dinickel molecular resonator

**DOI:** 10.1016/j.isci.2026.117076

**Published:** 2026-08-06

**Authors:** Miao Meng, Ying Ning Tan, Yu Li Zhou, Zi Cong He, Zi Hao Zhong, Jia Zhou, Guang Yuan Zhu, Chun Y. Liu

**Affiliations:** 1 Department of Chemistry, College of Chemistry and Materials Science, Jinan University, 601 Huang–Pu Avenue West, Guangzhou 510632, China

**Keywords:** Jaynes-Cummings model, quantum optics, strong light-matter coupling, photon antibunching, Mollow triplet

## Abstract

Field quantization is fundamental to quantum optics and polaritonic chemistry and is usually explored in microcavities through light-matter interactions. Here, we report strong resonant coupling of the two-level excitation of a dinickel (Ni_2_) complex molecule with its intrinsically recycled scattering field in free space under ambient conditions using weak, off-resonant excitation. The Ni_2_ unit acts simultaneously as a Jaynes-Cummings (JC) molecular emitter and an angstrom-scale optical resonator. The system exhibits well-resolved resonance-fluorescence signatures, including vacuum Rabi doublets and Mollow triplets. Simulations of the second-order correlation function *g*^(2)^(*τ*) confirm photon antibunching and underdamped Rabi oscillations, consistent with JC dynamics. Collective coupling scales with molecular number *N* as $\Omega_{N}=N\sqrt{N}\Omega_{1}$, distinct from the $\sqrt{N}\Omega_{1}$ Tavis-Cummings scaling, enabling ultrastrong coupling with few molecules. These results demonstrate that a Ni_2_ complex can be a chemically defined molecular quantum emitter for nonclassical light generation and used for studying polaritonic control of chemical reactivity.

## Highlights

• Strong light-matter coupling in a single Ni_2_ molecule
• Cavity-free field quantization under ambient conditions
• *N*$\sqrt{N}$Ω scaling exceeding Tavis-Cummings model
• A molecular platform for Jaynes-Cummings dynamics

## Graphical abstract

**Figure:**
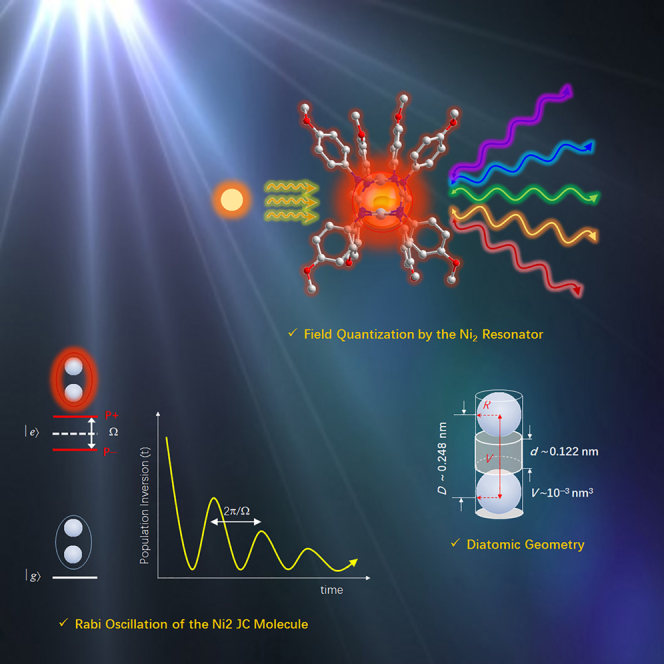

## Teaser

Physics; Optics; Materials science

## Introduction

In quantum optics, atoms and molecules act as canonical two-level emitters. Their ground state ∣*g*⟩ to excited state ∣*e*⟩ transition with a dipole moment *μ* interacts with a quantized electromagnetic field. The coherent coupling of a two-level excitation to the photonic states of a single optical mode forms a hybrid system characterized by two entangled dressed states—the exciton-polaritons.^1^^,^^2^^,^^3^ This physics is captured by the Jaynes-Cummings (JC) model,^4^^,^^5^^,^^6^ which predicts a ladder structure of dressed states with a vacuum Rabi splitting proportional to the coupling rate *g*.^7^ The coupling rate is governed by the transition dipole moment of the emitter and the strength of the driving field, which is inversely related to the effective photonic mode volume.^1^^,^^3^^,^^4^^,^^5^ To realize strong coupling, the emitter is typically driven by a resonant laser or placed inside a high-Q optical cavity. However, cavity minimization, which reduces the mode volume and thus enhances the emitter-cavity coupling, is approaching the technique limits in system construction and operation.

Recent advances in nanophotonics have enabled sub-nanometer plasmonic cavities, where minimizing the cavity size induces field-driven charge-transfer (CT) transitions between the two metal particles, producing plasmonic CT (PCT) modes.^8^^,^^9^ As the junction gap approaches the angstrom scale, quantum tunneling between metallic surfaces dominates.^10^ Recently, “picocavity” with ultralow mode volume (*V*_min_ ∼10^−2^ nm^3^) has been developed using a nanoparticle-on-mirror (NPoM) setup, enabling strong coupling at the single-molecule level.^11^ In the contact regime (*d* ≤ 0), the overlap of the atomic wavefunctions creates a covalent dimer that acts as an atomistic optical resonator, enabling CT transitions via quantum tunneling.^9^^,^^10^ The development of sub-nano plasmonic cavities suggests that appropriately designed dimetal (M_2_) complex molecules with a M–M separation of few angstroms can function as intrinsic resonators, supporting quantum-optical interactions without the need for externally constructed cavities and realizing strong coupling between the molecule and the extremely confined field.

In addition to reducing the photonic mode volume, collective coupling provides another route to enhance light-matter interaction strength.^1^^,^^3^^,^^12^^,^^13^^,^^14^ The Tavis-Cummings (TC) model^12^ predicts that coupling *N* identical emitters to a single-photon field yields a collective Rabi frequency Ω_TC_ = $\sqrt{N}$Ω_1_.^3^^,^^13^ When a few JC-type molecules are simultaneously but individually driven by quantum light of the same mode, the ensemble would behave as an effective “JC molecular system” with total dipole moment *N*μ. In this configuration, simultaneous excitation of *N* molecules (ω_0_) by absorbing *N* scattered photons (ω_L_ ≈ ω_0_) is expected to result in a collective coupling strength Ω_*N*_ = *N*$\sqrt{N}$Ω_1_, exceeding the $\sqrt{N}$ enhancement predicted by the TC model. This distinguishes the JC-molecule ensembles from Dicke-type collective excitations involving symmetric superposition states,^12^^,^^15^ which, however, has not been experimentally demonstrated.

**Figure 1:**
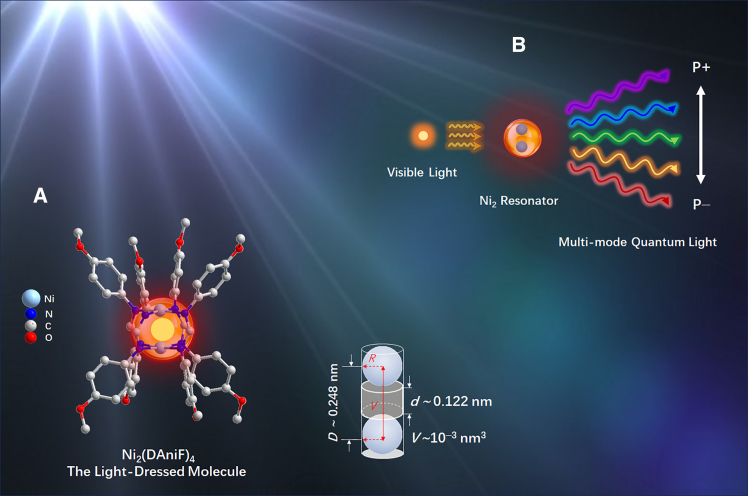
Geometric structure and scattering-field response of the Ni_2_ resonator (A) Structural representation of Ni_2_(DAniF)_4_, showing the Ni_2_ dimer embedded within the ligand framework. The inset shows the angstrom-scale Ni···Ni separation (2.48 Å) modeled as a cylindrical resonator, which defines the ultralow effective mode volume on the order of 10^−3^ nm^3^. (B) Pictorial illustration of field quantization by the Ni_2_ resonator. In this study, the Ni_2_ unit converts a broadband classical pump field into discrete scattering modes, producing multiple quantized photonic channels via the JC dressed-state manifolds.

In this study, we investigated the dinickel (Ni_2_) complex Ni_2_(DAniF)_4_ (DAniF, *N*, *N*′- di(*p*-anisyl)formamidinate) in terms of light-matter interaction. In this complex molecule, the Ni_2_ unit is composed of two unbonded Ni^2+^ ions separated by approximately 2.5 Å, as shown in Figure 1A, and could serve as a diatomic optical resonator, in analogy to a nanoparticle cavity at the angstrom scale. We found that the Ni_2_ molecule functions as a diatomic JC emitter and that the Ni → Ni charge transition (ω_0_) is coherently coupled with the scattered photons from the Ni_2_ resonator under ambient, cavity-free conditions. Strong and ultrastrong coupling in single molecules and the *N*-molecule ensembles produces resonant fluorescence featuring a scattering continuum with discrete photonic modes that correspond to the exciton-polaritons, as pictorially illustrated in Figure 1B. The results verify the *N*$\sqrt{N}$Ω_1_ scaling for collective coupling in an effective JC system (*N* = 2 or 3). The Ni_2_ system provides a clearer spectral resolution of the optical responses observed in the Mo_2_ complexes in a prior study,^16^ allowing quantitative evaluation of the JC-type scaling and collective coupling for *N* = 1–3.

## Results

### Experimental and theoretical models

The Ni_2_(DAniF)_4_ molecule under investigation consists of a Ni_2_^4+^ core that is equatorially coordinated by four DAniF ligands (Figure 1A). The molecular and electronic structures, as well as the absorption spectra, have been characterized previously from a chemical standpoint.^17^^,^^18^ Structurally, Ni_2_ formamidinates comprise two square-planar Ni^2+^ centers without formal metal-metal bonding (Figure 1A). X-ray diffraction measurements show a Ni···Ni separation of 2.48(5) Å.^18^ The Ni_2_ unit can be modeled as a cylindrical, angstrom-scale resonator (Figure 1A, inset). Using an interatomic distance *D* = 0.248 nm and ionic radius *R* = 0.063 nm,^19^ the effective interfacial distance becomes *d* = 0.122 nm. From this geometry, the free-space volume confined between the two Ni centers is estimated to be ∼10^−3^ nm^3^, which is one order of magnitude smaller than the minimum mode volumes reported for plasmonic picocavities.^11^ This extremely limited mode volume implies a tremendous enhancement of the local electric field, enabling strong coupling in the absence of an external cavity.

**Figure 2:**
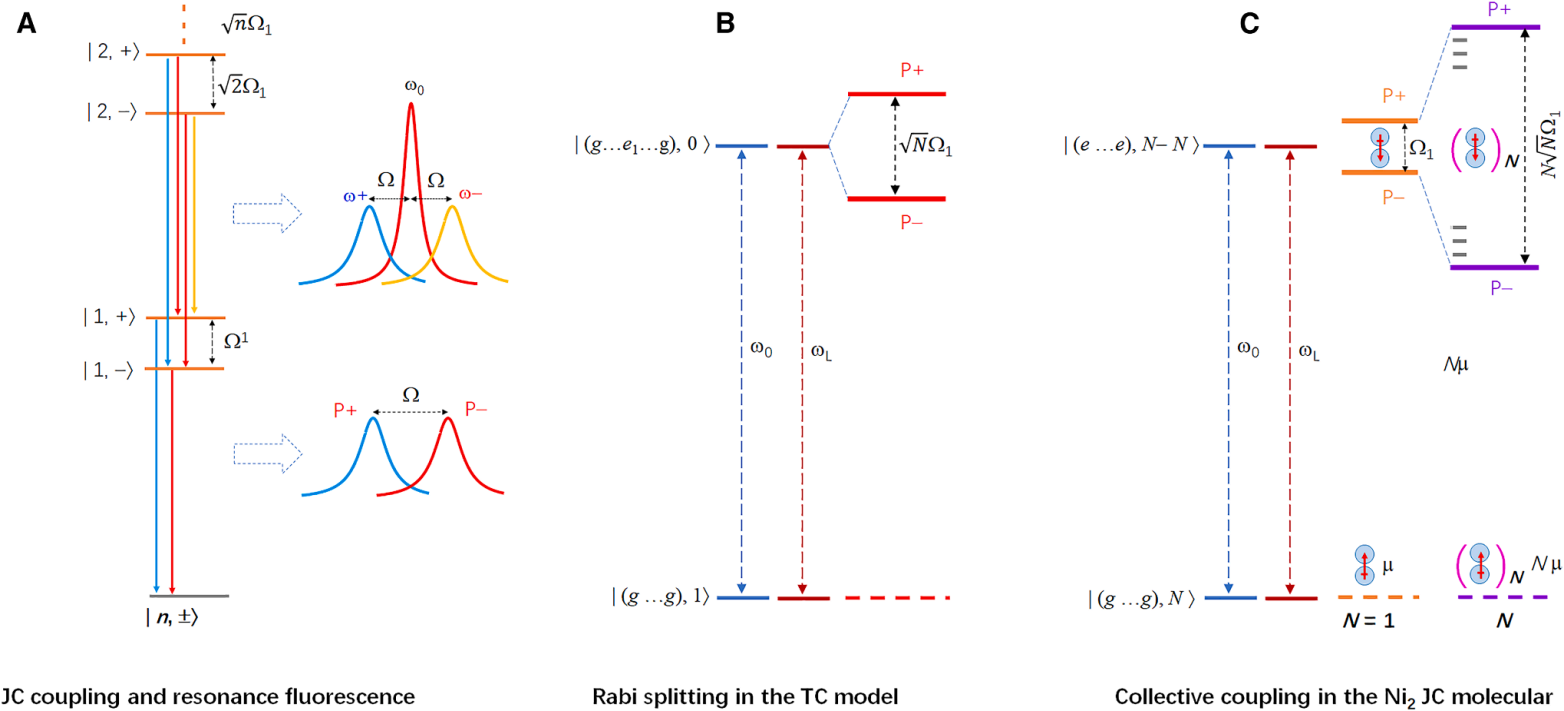
Modification of molecular eigenstates through resonant coupling with an electromagnetic field (A) A schematic of Jaynes-Cummings (JC) coupling between a two-level excitation and photon-number states |*n*⟩, producing the vacuum Rabi doublet and Mollow triplet characteristic of resonance fluorescence. The dressed-state ladder illustrates the energy-level progression with increasing photon number *n*. (B) Collective coupling predicted by the Tavis-Cummings (TC) model, in which the vacuum Rabi splitting scales as Ω_TC_ = $\sqrt{N\;}$Ω_1_ for *N* identical emitters coherently coupled to a single field mode. (C) Collective coupling in the Ni_2_-JC molecular system, where *N* independently driven molecules with total dipole moment *N*μ interact with *N* scattered photons, producing a nonlinear collective Rabi splitting that scales as *N*$\sqrt{N}$Ω_1_, *N* times larger than the $\sqrt{N}$Ω_1_ scaling in the TC model. The upper and lower gray levels represent the P+ and P− exciton-polaritons, respectively, for ensembles with molecular numbers increasing stepwise to *N* from 1.

The strong coupling of a two-level excitation (ω_0_) with a quantized optical field in a single molecule can be described by the JC Hamiltonian,

(Equation 1) $$H_{\text{JC}}=\omega_{L}a^{†}a+\omega_{0}\sigma_{z}+g\left(a\sigma_{+}+a^{†}\sigma_{-}\right)\text{,}$$

where *a*^†^ and *a* are the photon creation and annihilation operators of the relevant optical mode, and σ_±_ and σ_z_ are the Pauli operators of the molecular two-level transition. The coupling rate *g* gives the single-molecule vacuum Rabi splitting Ω_1_ = 2*g*. The JC physics predict the spectral structure of the driven molecule, known as the JC molecule, which features a Rabi doublet with two components^1^^,^^2^^,^^3^^,^^6^ at ω_0_ ± *g* or a Mollow triplet^20^^,^^21^^,^^22^ with the central peak at ω_0_ and two sidebands at ω_0_ ± Ω (Figure 2A). In the present Ni_2_ complex, the two-level transition is assigned to the interatomic CT excitation of the Ni_2_ unit, while the optical mode corresponds to the locally recycled scattering field (ω_L_) with ω_L_ ≈ ω_0_ confined by the molecular resonator.

For an ensemble of *N* identical Ni_2_ molecules, the conventional TC model would describe *N* emitters coupled to one externally supplied cavity mode. The interaction term *H*_int_ = *g*(*aJ*_+_ + *a*^†^*J*_−_), where $J_{\pm }=\sum_{j=1}^{N}\sigma_{\pm ,j}$, giving the usual $\sqrt{N}$ enhancement of the collective transition amplitude. The Ni_2_ system considered here is different. Each Ni_2_ molecule acts not only as a two-level emitter but also as a local molecular resonator that contributes coherently to the recycled scattering field. Therefore, an *N*-molecule ensemble is treated as an effective collective JC molecular-resonator system rather than as a standard TC ensemble.

Under this assumption, the interaction term contains two coherent summations: one over the molecular field contributions and the other over the molecular transitions. We write the effective Hamiltonian as

(Equation 2) $$H_{N\text{JC}}=\sum_{\mu =1}^{N}\omega_{L}a_{\mu }^{†}a_{\mu }+\sum_{j=1}^{N}\omega_{0}\sigma_{z,j}+g\sum_{\mu =1}^{N}\sum_{j=1}^{N}\left(a_{\mu }\sigma_{+,j\;}+a_{\mu }^{†}\sigma_{-,j\;}\right)\text{,}$$

where *a*_*μ*_ denotes the local scattering-field contribution from the *μ*-th molecular resonator, and σ_±,*j*_ and σ_z,*j*_ describe the two-level transition of the *j*-th molecule. The double summation in the interaction term expresses the effective all-to-all coupling between the collective molecular scattering field and the molecular transitions. This form assumes that the participating molecular resonators are transiently phase matched and that their scattered fields add coherently over the lifetime of the coupled state.

We then introduced the normalized collective field operator and the collective molecular spin operators,

(Equation 3) $$A=\frac{1}{\sqrt{N}}\sum_{\mu =1}^{N}a_{\mu },J_{z}=\sum_{j=1}^{N}\sigma_{z,j},J_{\pm }=\sum_{j=1}^{N}\sigma_{\pm ,j}\text{.}$$

The collective field mode *A* corresponds to the symmetric bright mode obtained from a unitary transformation of the *N* local scattering-field contributions.^23^^,^^24^ It satisfies the standard bosonic commutation relation [*A*,*A*^†^] = 1. Keeping only this bright collective field mode and neglecting dark field combinations,^24^ the interaction term becomes

$$g\sum_{\mu =1}^{N}\sum_{j=1}^{N}a_{\mu }\sigma_{+,j}=g\left(\sum_{\mu =1}^{N}a_{\mu }\right)\left(\sum_{j=1}^{N}\sigma_{+,j}\right)=\sqrt{N}gAJ_{+}\text{,}$$

including the Hermitian-conjugate term that gives the effective collective JC interaction,

(Equation 4) $$H_{\mathrm{int}}^{\text{eff}}=\sqrt{N}g\left(AJ_{+}+A^{†}J_{-}\right)\text{.}$$

Thus, the effective Hamiltonian in the bright collective subspace is

(Equation 5) $$H_{\text{eff}}=\omega_{L}A^{†}A+\omega_{0}J_{z}+\sqrt{N}g\left(AJ_{+}+A^{†}J_{-}\right)\text{.}$$

Here, the prefactor $\sqrt{N}g$ arises from the coherent summation of the *N* molecular scattering-field contributions.^23^^,^^24^ This field-originated enhancement is not present in the conventional TC Hamiltonian, where the ensemble couples to a single pre-existing cavity mode rather than to a field generated collectively by molecular resonators.

The collective Rabi splitting is obtained from the effective coupling amplitude in the symmetric collective subspace. The collective molecular transition described by *J*_±_ contributes the usual $\sqrt{N}$ enhancement of the transition amplitude, as in the TC model,^12^^,^^13^ while occupation of the collective field mode by *n* photons contributes the bosonic factor $\sqrt{n}$. Therefore, the effective coupling amplitude is

(Equation 6) $$g_{N}\left(n\right)=\sqrt{N}g\times \sqrt{N}\times \sqrt{n}=Ng\sqrt{n}\text{.}$$

For the effective JC molecular system considered here, simultaneous excitation of *N* identical molecular resonators is accompanied by an effective occupation of *n* = *N* scattered/recycled photons in the collective mode. This gives

$$g_{N}=N\sqrt{N}g\text{,}$$

and hence,

(Equation 7) $$\Omega_{N}=2g_{N}=2N\sqrt{N}g=N\sqrt{N}\Omega_{1},\Omega_{1}=2g\text{,}$$

where Ω_*N*_ is the effective vacuum Rabi splitting of the *N*-molecule ensemble at ω_0_.

### Optical responses of the Ni_2_ complex to weak non-resonant excitation

**Figure 3:**
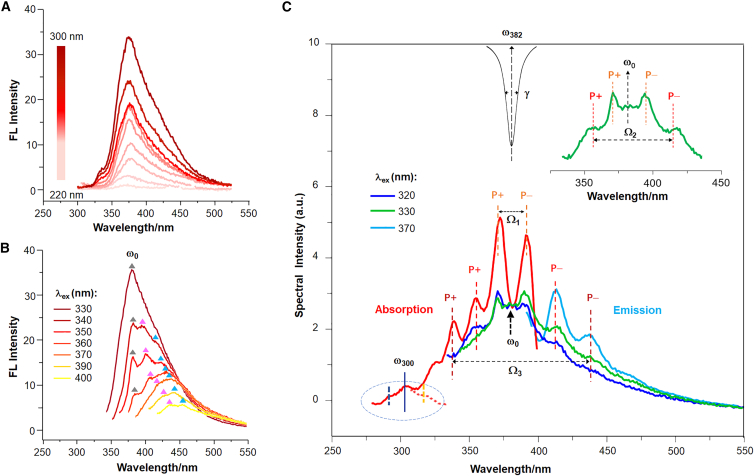
Optical responses of Ni_2_(DAniF)_4_ under weak, non-resonant excitation (A) Fluorescence spectra under far-off resonant excitation (λ_ex_ = 220–300 nm), showing a thermally averaged scattering field characteristic of photon bunching. (B) Excitation at 330–400 nm resolves a sharp Rayleigh peak at 382 nm and several red-shifted sidebands, indicating discrete, phase-coherent scattering modes associated with the Ni_2_ emitter. (C) Combined absorption and emission spectra revealing the Rabi doublets (R) and Mollow sidebands (M) for *N* = 1, 2, and 3. Vertical dashed (R) and solid (M) lines indicate the experimentally identified dressed states. The circled region highlights the Mollow triplet at 300 nm with two sidebands at (300 ± Ω_1_′). Inset: overlaid spectra (320–330 nm excitation) resolving P+ and P− polaritons for *N* = 1 and 2. Figure S2 schematizes the simulated polaritonic transitions in the single and multiple photon processes.

Ni_2_(DAniF)_4_ in dichloromethane (DCM) solution exhibits a broad, asymmetric emission band with a maximum at ∼380 nm when excited between 220 and 300 nm (Figure 3A). This feature does not correspond to any known intramolecular electronic transition of the complex,^17^^,^^18^ meaning that the emission is not governed by the conventional 0–0 relaxation pathway. Instead, this fluorescence energy falls in the range for high-order PCT transition (∼3 eV) for nanoparticle dimers in the quantum contact regime (*d* < 0),^9^ supporting a CT transition between the two metal atoms. The transition energy for the Ni_2_ complex is similar to those for Cu_2_(O_2_CR)_4_ (420 nm)^16^ and Mo_2_(O_2_CCH_3_)_4_ (400 nm),^25^ which differ in electronic structure. This suggests that the optical response is dominated primarily by the M–M geometry of the M_2_ unit, analogous to the dependence of PCT modes on junction separation in metallic nanoparticle dimers.^9^^,^^10^^,^^11^ These observations imply that the broad emission arises from an interatomic CT (CT_0_) transition occurring in the quantum tunneling regime (*d* < 0),^9^ where decreasing metal-metal separation increases the CT transition energy.^10^ The spectral profiles suggest that under far-off-resonant excitation, the scattered field is thermally averaged, producing a featureless fluorescence band that indicates photon bunching (Figure 3A).

As the excitation wavelength approaches the CT_0_ resonance, the broad emission evolves into a set of discrete, narrow optical modes, with a high-energy peak fixed at 382 nm and additional red-shifted sidebands (Figure 3B). The 382 nm peak is assigned to the elastic Rayleigh scattering from the Ni_2_ unit, as its position remains invariant with pump energy. This transition defines the operative CT two-level excitation (ω_0_) of the molecule as a JC emitter. By deconvolution of the 350-nm-excited spectrum (Figures 3B and S1A), the natural linewidth γ (FWHM ≈1,000 cm^−1^) of the ω_0_ transition is extracted, providing a fundamental parameter for the coherent dynamics. The lower energy sidebands systematically red-shift as the excitation energy decreases, reflecting phase-coherent coupling between the molecular excitation and the scattered field. This transformation from thermal averaged scattering to discrete modes is understandable from the characteristics of resonance fluorescence for the two-level Ni_2_ molecule,^26^ demonstrating quantization of the local scattering field. Thus, the Ni_2_ unit plays the role of a diatomic resonator with the nature frequency ω_L_ ≈ ω_0_.

### Vacuum Rabi splitting of ω_0_ induced by non-resonant excitation for single and collective coupling

Under weak, non-resonant monochromatic excitation (λ_ex_ = 320–330 nm), a dilute solution of Ni_2_(DAniF)_4_ exhibits a characteristic three-line fluorescence structure. The central peak is located at 382 nm, with two symmetric sidebands at 372 and 392 nm (Figure 3C). The central feature is narrower and weaker than the sidebands, consistent with its assignment as Rayleigh scattering at the resonance frequency (ω_0_). The sideband separation, Ω_1_(exp) = 1,380 cm^−1^, satisfies the condition Ω ≫ γ/4 (Figures S1), confirming that the system is in the strong-coupling regime with a resolvable three-line resonance-fluorescence spectrum.^27^^,^^28^ Therefore, the two sidebands are assigned to the upper (P+) and lower (P−) exciton-polaritonic branches for the dressed single molecule with a coupling strength Ω_1_. The invariance of peak positions with pump wavelength indicates that the coupling strength is intrinsic to the Ni_2_ resonator rather than determined by excitation conditions or wavelength of the incident light. An additional pair of peaks appears at 356 and 413 nm (Figure 3C, inset), which is attributed to the Rabi doublet (P+ and P−) for the two-molecule ensemble. Their splitting of 3,875 ± 10 cm^−1^ matches the expected value for *N* = 2 collective coupling according to the scaling law *N* $\sqrt{N}$ Ω_1_ (Equation 7). The blue sidebands from higher order collective coupling, which are typically inaccessible under off-resonant excitation, can nevertheless be observed in the absorption spectrum using appropriate excitation.^29^ The combined excitation (emitted at 418 nm) and emission (excited at 370 nm) spectra (Figure 3C) confirm the polaritonic states for systems with *N* = 1 and 2 observed in the resonance fluorescence and further resolve the JC coupling for *N* = 3. For the three-molecule ensemble, the absorption peak at 336 nm and emission peak at 442 nm flank ω_0_ with a measured splitting of 7,138 ± 20 cm^−1^, in excellent agreement with Ω_3_ = 7,120 cm^−1^ calculated from the $N\sqrt{N}$ Ω_1_ scaling. These two peaks are therefore assigned to the P+ and P− branches of the *N* = 3 manifold. For the single-molecule JC system, the normalized coupling strength is η_1_ = g/ω_0_ = 0.026, consistent with strong exciton-field coupling (η > 0.01).^14^ For *N* = 3, η_3_ = 0.14 places the system in the ultrastrong-coupling regime (η > 0.1).^12^^,^^14^ These results demonstrate that the Ni_2_-JC emitter efficiently facilitates strong and ultrastrong coupling with rotating-wave approximation (RWA).^14^

**Figure 4:**
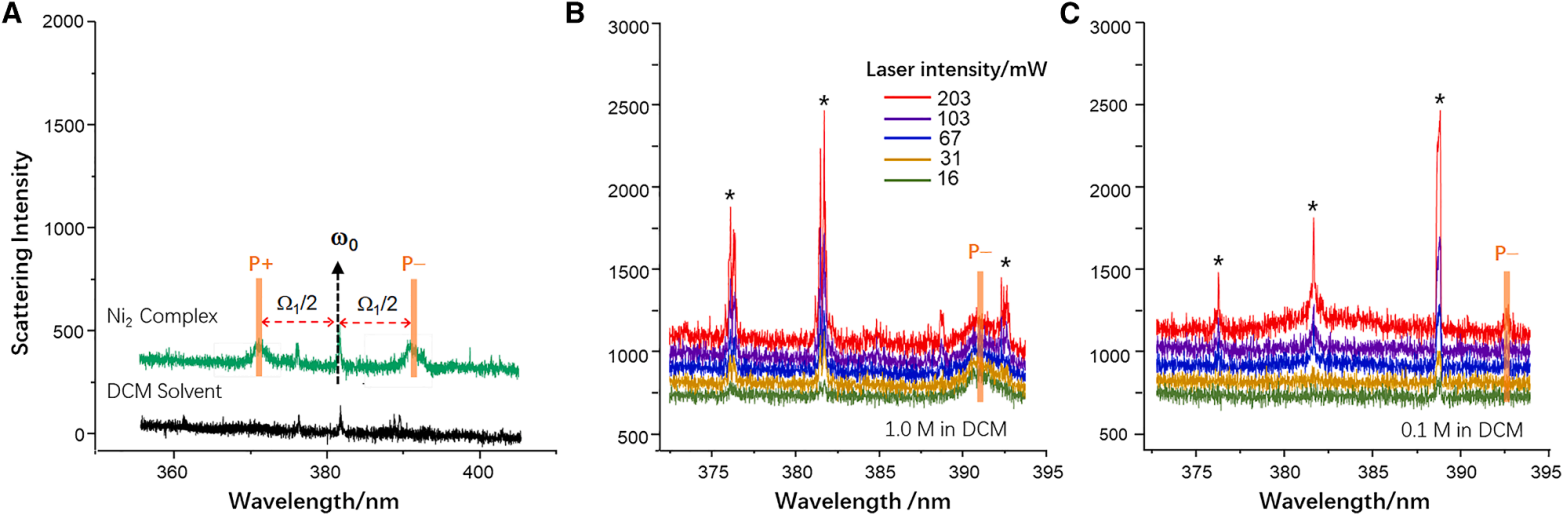
Scattering spectra and power/concentration dependence for Ni_2_(DAniF)_4_ excited using 325-nm continuous-wave laser The asterisk marked signals are the optical background of the laser beam. (A) Scattering spectrum showing the Rabi-split peaks at 372 (P+) and 392 nm (P−) with separation Ω_1_ = 1,380 cm^−1^, confirming that coupling is driven by the intrinsic scattering field, rather than by the incident laser. (B and C) Scattering spectrum for the concentrated (1.0 M) and dilute (0.1 M) solutions, respectively, excited at varying laser powers. The unchanged Rabi state (P−) at ∼392 nm across all power levels confirms that the local field ω_L_ is coherently coupled to the CT_0_ frequency (ω_0_).

To verify that the coupling is mediated by the intrinsic scattering field rather than the incident light, Ni_2_(DAniF)_4_ was illuminated with a high-intensity, 325 nm continuous-wave laser (Figure S3). The resulting scattering spectrum (Figure 4A) shows two broad peaks at ∼372 and ∼392 nm, separated by 1,380 cm^−1^, identical to the single-molecule splitting extracted from the resonance fluorescence under weak excitation (Figure 3C). Importantly, varying the laser power does not alter the peak positions, though higher intensities are required for dilute samples (Figures 4B and 4C). This invariance in transition energy demonstrates that the light-matter interaction is driven by the recycled scattering field at ω_0_, confirming that the Ni_2_ unit operates as an angstrom-scale optical resonator affording an intrinsically enhanced scattering field.

### Time-domain correlation functions *g*^(2)^(τ) and coupling mechanism for single and collective coupling

**Figure 5:**
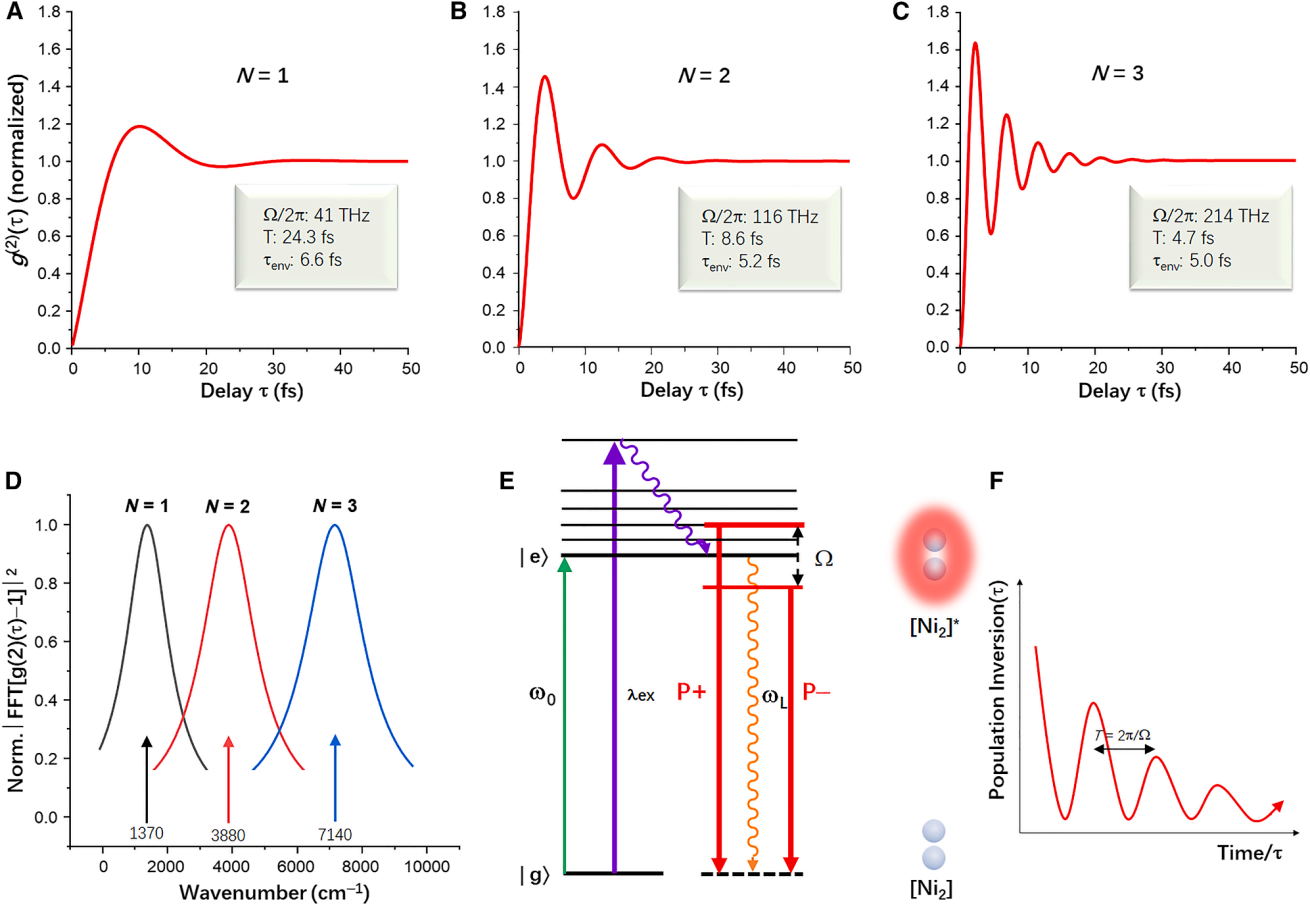
Time-domain photon-correlation functions and coupling mechanism for the *N* = 1–3 JC systems (A–C) Simulated *g*^(2)^(τ) functions for *N* = 1, 2, and 3 using the Carmichael-Walls (CW) three-line model. Insets list the fitted parameters (Ω/2π, T, and τ_env_). *g*^(2)^(0) = 0 indicates photon antibunching, while *g*^(2)^(τ) oscillations at τ > 0 reflect coherent Rabi dynamics of the dressed JC states. (D) Frequency-domain *g*^(2)^(τ) representations, obtained by Fourier transform of the calculated time-domain *g*^(2)^(τ) traces for the three systems (*N* = 1, 2, and 3). The common linewidths (FWHM ≈1,500 cm^−1^), determined by the radiative damping parameter (γ = 1,000 cm^−1^), were used in the simulations. (E) Schematic of resonant coupling between the CT_0_ transition (ω_0_) and the local scattering field (ω_L_) stemming from non-resonant excitation. (F) Representation of Rabi oscillation in the Ni_2_ emitter excited by classical light and dressed by the recycled scattering field.

The second-order correlation function *g*^(2)^(τ) for resonance fluorescence in strongly driven two-level systems is well established theoretically^27^ and has recently been demonstrated experimentally at the single-molecule level.^22^ Based on the JC formalism^4^^,^^5^ and Carmichael’s quantum trajectory method,^26^ we simulated *g*^(2)^(τ) for the three Ni_2_-based coupling configurations (*N* = 1, 2, and 3) using the experimentally extracted spectral parameters. Simulations were performed using the Carmichael-Walls (CW) three-line model in the one-photon limit (Equation 8, *n* = 1),^27^^,^^30^

(Equation 8) $$g^{\left(2\right)}\left(\tau \right)={\left[g^{\left(1\right)}\left(0\right)\right]}^{2}\left[1-\exp\left(-\left(3/4\right)\gamma \tau \right)\cos\left(\sqrt{n}\;\Omega \tau \right)\right]\text{,}$$

where γ is approximated by the averaged sideband linewidth (FWHM) and τ_env_ = 3/4γ for the strong-drive envelope. Simulations produced oscillatory behavior and characteristic decay parameters for each system (Figures 5A–5C), validating their applicability across the coupling regimes. For *N* = 1, the oscillation frequency extracted from *g*^(2)^(τ) matches the measured Ω_1_ = 1,380 cm^−1^, and the simulations yield decay parameters including the oscillation period *T* and envelope decay time τ_env_ (inset values in Figures 5A–5C). The value *g*^(2)^(0) = 0 indicates photon antibunching,^25^^,^^31^^,^^32^ corresponding to spontaneous emission of the first photon scattered from the Ni_2_ core that initiates the Rabi oscillation (Figure 5F). These systems exhibit photon correlations that differ from those for undriven or weakly interacting two-level molecules, where photon antibunching is manifested by the exponential intensity decay at resonance.^26^^,^^30^^,^^33^ The oscillatory *g*^(2)^(τ) behavior at τ > 0 reflects the coherent evolution of the scattered field. These results are in agreement with the dynamical responses of the field for τ > 0 under the condition on a photon detection at τ = 0 in cavity-QED (quantum electrodynamics) systems under weak driving (photon antibunching).^34^ Thus, simulations from the CW model (Equation 8)^27^ explain the three-line fluorescence spectra for the JC systems (Figure 3C): the narrow central line corresponds to Rayleigh scattering at ω_0_, while the sidebands are positioned at ω_0_ ± *g*_*N*_, where *g*_*N*_ = $\frac{1}{2}\left(N\sqrt{N}\Omega \right)$ (*N* = 1, 2, and 3). Fourier transformation of the simulated *g*^(2)^(τ) traces (Figure 5D) yields the frequency-domain spectra that reproduce the experimentally observed Rabi splittings for the three systems. Therefore, the simulations of *g*^(2)^(τ) confirm that the Ni_2_ resonance-fluorescence dynamics are quantitatively described by the CW and JC formalisms and that the calculated nonclassical correlations arise from resonant coupling between the CT_0_ excitation at ω_0_ and the locally recycled scattering field (Figure 5E). In light of this, the *N*-molecule ensemble can be viewed as a “large JC molecule,” with the transition frequency remaining at ω_0_ and an effective collective dipole moment *Nμ*, which is coupled to *N* scattered photons in accordance with the effective Hamiltonian (Equation 5).

### Quantization of classical light and phase transition of the nonclassical field

**Figure 6:**
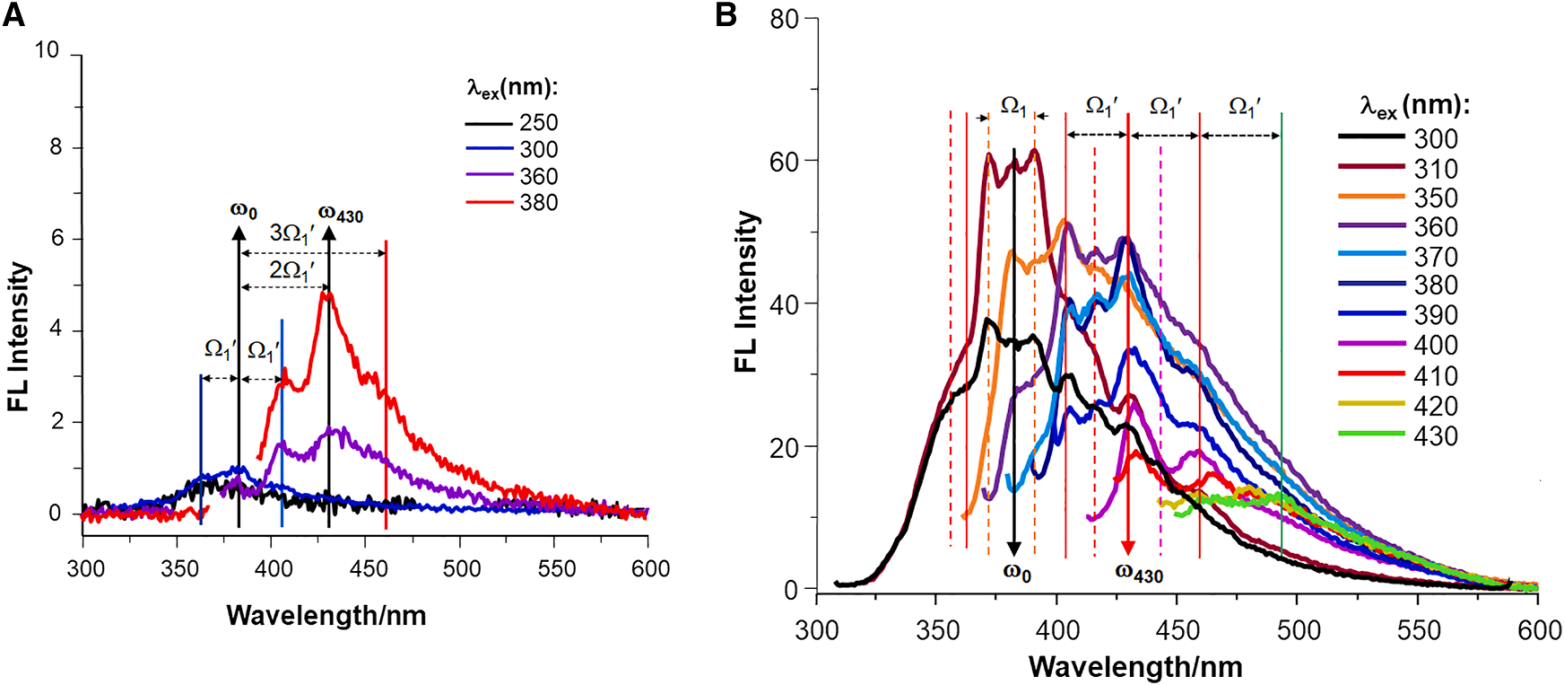
Quantization of the scattering field and excitation-induced phase transitions (A) Fluorescence spectra showing the emergence of the Mollow triplets and the higher order sidebands under varying excitation wavelengths. Peaks at 360, 380, and 402 nm characterize the Mollow triplet at resonance (ω_0_) with two satellites at ω_0_ ± Ω_0_′, while the 430 nm triplet represents the second-order Mollow sideband that is resonantly coupled to the scattered photons from the (ω_0_ − 2Ω_0_′) state for *N* = 2. (B) Evolution of Rabi (R) and Mollow (M) transitions for *N* = 1–3 by lowering the excitation energy. Adjacent R and M peaks are separated by ∼700 cm^−1^, indicating Ω_1_/2 or π-phase shifts between the successive dressed states. Excitation at 310–360 nm induces phase-controlled population transfer between the Rabi and Mollow manifolds. Additional sidebands at 404–530 nm arise from collective sideband excitation across the JC and Mollow states.

For a concentrated solution of Ni_2_(DAniF)_4_ (C = 0.1 mM), weak, high-energy excitation (e.g., λ_ex_ = 250 nm) yields an extremely weak ω_0_ peak accompanied by barely observable sidebands (Figure 6A, black trace). Upon dilution to 0.0125 mM and excitation at 300 nm, the three-line structure becomes well resolved at 360, 380, and 402 nm (Figure 6A, blue trace), characteristic of the Mollow triplet centered at ω_0_.^20^^,^^21^ Two sidebands appear at ω_0_ ± Ω_1_′, where Ω_1_′ = 1,450 cm^−1^, reasonably larger than Ω_1_ from vacuum Rabi splitting (1,380 cm^−1^). Presumably, this Ω_1_′ results from the multi-photon excitation enabled by photon bunching in the far-off-resonant regime (Figure 3A). The observed asymmetric intensity distribution and broadened central peak are attributed to the incoherent excitation, and the spectral distortion from the standard Mollow triplet is likely due to the squeezed local field of a JC system.^35^^,^^36^ At lower excitation energies, three peaks appear at 404, 430, and 458 nm (Figure 6A, purple). These features correspond to additional low-energy Mollow-type sidebands, denoted ω_0_ − *N*Ω_1_′ (*N* = 1, 2, …), associated with the collective JC coupling (Figure S4). Notably, the increased intensity of the second sideband for *N* = 2 has been theoretically predicted for the few-emitter JC systems.^37^^,^^38^ Similar families of sidebands have been observed in *N*-atom Rb ensembles, where the separation between the outer sidebands increases as *N* increases.^39^ Under resonant excitation at 380 nm, the fluorescence evolves into a strong Mollow triplet centered at 430 nm (Figure 6A, red). The appearance of the ω_430_ Mollow triplet can be understood from the increased scattering field at the second red sideband (ω_0_ − 2Ω_0_′) at resonant pumping for the *N* = 2 collective system.^36^ This dressed two-molecule ensemble is able to provide a multi-photon leapfrog at the Mollow triplet sideband,^21^ enabling the two-photon process for mode-selective excitation at ω_430_ and demonstrating the so-called Mollow spectroscopy proposed recently.^40^

Upon further dilution (C = 0.1 μM), the fluorescence intensity increases notably (Figure 6B). This trend supports the interpretation that the molecule-field interaction is driven by the scattered field. This is because photon antibunching is suppressed^36^^,^^39^ at higher concentrations where intermolecular interactions induce photon bunching^41^ and suppress coherent coupling pathways. Under 300 nm excitation, the spectrum reveals the Rabi doublets and Mollow sidebands for *N* = 1, 2, and 3 (Figure 6B, black), forming a staircase-like progression. The adjacent Rabi doublet (R) and Mollow triplet (M) peaks are separated by approximately 700 cm^−1^, corresponding to Ω_1_/2 or (Ω_1_′ − Ω_1_/2), indicating a phase relationship between successive dressed states. Excitation at 310 nm produces intense P+ (372 nm) and P− (392 nm) polaritons (*N* = 1) and a weak ω_0_ peak (Figure 6B, dark red trace), consistent with the emission spectra excited at 320–330 nm (Figure 3C, inset). At 350 nm excitation, the ω_0_ and 404 nm Mollow states dominate in the spectrum, while the 392 nm peak is diminished. This spectrum appears as if the 310-nm-excited profile is red-shifted by Ω/2 (π phase), indicating an excitation-induced phase transition between the Rabi and Mollow states for *N* = 1. Lowering the excitation energy to 360 nm induces an additional π phase shifting, producing peaks at 404 and 430 nm centered around 415 nm, assigned to the P− branch of the *N* = 2 system. Compared to the spectrum in Figure 3C, the intensity of this 415 nm emission is largely reduced due to the sideband excitation. The fluorescence lineshapes and splittings resulting from sideband excitations are identical to those observed for resonant coupling at ω_0_ under non-resonant excitations, indicating that the underlying two-level transition is resonantly coupled to the scattered field regardless of the excitation path. These phase-controlled spectra confirm that successive π/2 phase shifts transform the Rabi-dominated emission into the Mollow-dominated emission, revealing coherent modulation over dressed-state populations. Such behavior supports that sideband excitation through photon antibunching provides an effective excitation trajectory,^39^ enabling access to higher order polaritonic states. These results demonstrate that resonance fluorescence can be used as a novel source of nonclassical light in the study of quantum optics^25^^,^^26^^,^^32^^,^^39^ and in the development of quantum technologies.^41^ In particular, this Ni_2_ system offers the advantages in weak, non-resonant excitation and cavity-free, ambient operating conditions.

Under near-resonant excitation (370 nm), the fluorescence intensity at 382 nm is minimized, forming a deep spectral “hole” (Figure 6B), consistent with the spectral valley observed at ω_0_ in the excitation-emission map (Figure 3C). This suppression of the scattered field at ω_0_ manifests resonant coupling of the molecular excitation to the scattering field.^22^^,^^31^ The spectra excited at 370–400 nm reveal the evolution of the ω_430_ Mollow triplet (Figure 6B), consistent with the spectra (Figure 6A) for the concentrated solution. Additional sidebands, ω_0_ − *N*Ω_1_′, appear at 490 nm (*N* = 4) with λ_ex_ = 430 nm (Figure 6B) and 528 nm (*N* = 5) under 450 nm excitation (Figure S4). Their blue counterparts (ω_0_ + 4Ω_1_′) and (ω_0_ + 5Ω_1_′) are observed as the red sideband (ω_300_ − Ω_1_′) and the central line for the Mollow triplet at 300 nm, respectively, in the excitation spectrum (Figure 3C, circled area). Formation of additional sidebands at twice the frequency of the classical Mollow sidebands has also been observed recently in the fluorescence spectrum of an atomic cloud at low density that scatters classical light.^42^ In this Ni_2_ system, the high-order sidebands result from polariton excitation of the collective Mollow transitions,^39^ which confirms phase coherence across multiple JC systems.^22^ Notably, the same 430 nm triplet and its sidebands have been observed in Mo_2_(DAniF)_4_^16^ and other dimolybdenum complexes,^24^ indicating that the M_2_ systems with similar metal-metal separations (2.1–2.3 Å) exhibit virtually identical quantum-optical dynamics, regardless of their electronic structures.

Furthermore, in solution, molecular motion and stochastic orientation would normally broaden or obscure such coherent features. However, in this Ni_2_ system, the spectra consistently show highly homogeneous polaritonic signatures without contributions from the uncoupled electronic transitions, demonstrating that all molecules are dressed upon weak excitation. The collective coupling behavior can be attributed to the transient formation of few-molecule ensembles (*N* = 2 and 3) with parallel dipole orientations, enabling collective coupling through the shared scattering fields. The absence of higher order (*N* > 3) features can be explained by the low probability of forming larger aligned ensembles in solution. In addition, the JC-type scattering is known to generate squeezed states,^34^^,^^35^ which relax the constraints of dipole blockade, facilitate collective excitation beyond the Dicke limit,^43^ and enable diminishing the optical noise.^25^

## Discussion

Traditionally, achieving resonant light-matter coupling requires high-Q optical cavities, cryogenic operation, and intense resonant driving. In contrast, the Ni_2_ molecular system exhibits measurable vacuum Rabi splitting, Mollow triplets, and quantized scattering modes under ambient, cavity-free, and weakly driven conditions. The splitting of the CT_0_ molecular resonance at 382 nm (ω_0_) under non-resonant excitation, together with simulations of the second-order correlation function *g*^(2)^(τ), demonstrates that individual Ni_2_ molecules strongly couple to their intrinsically confined local scattering field. Analysis of the *N*-molecule JC Hamiltonian, combined with detailed spectral examinations, shows that the dressed Ni_2_ molecules function as individual JC emitters and that collective coupling emerges for transient ensembles of *N* molecules (*N* = 2 and 3). The collective Rabi frequencies follow Ω_*N*_ = *N* $\sqrt{N}$ Ω_1_, *N* times larger than the $\sqrt{N}$ enhancement predicted by the TC model. The Ni_2_ system produces a quantized scattering field with discrete single modes in a broad range of wavelengths, as evidenced by the fluorescence ladder and higher order Mollow sidebands. These observations confirm that the field granularity originates from the JC and Mollow manifolds of the Ni_2_ resonator rather than from an external cavity.

In this Ni_2_ system, mode-selective excitation is achieved via classical-light absorption or non-resonant pumping. The quantized scattering field exhibits nonclassical photon correlations and behaves analogously to the transmitted field in cavity-QED systems.^33^ In the resonance fluorescence spectra, the thermally averaged scattering continuum (Figure 3A) is resolved into discrete single photonic modes, achieving field quantization (Figure 3B). Unlike microcavity systems where quantization is imposed externally,^1^^,^^2^ here field granularity arises intrinsically from the JC and Mollow manifolds of the Ni_2_ resonator, enabling quantization of classical light using steady-state spectroscopy under ambient conditions.

This Ni_2_ molecular system exhibits a set of unusual and technologically attractive quantum-optical properties, including strong and ultrastrong light-matter coupling, cavity-free driving under weak (or strong) non-resonant excitation, robust light-matter hybridization, and field quantization under ambient conditions in solution. As a chemically defined and chemically programmable molecular platform, it combines quantum functionality with the tunability of synthetic coordination chemistry. These features make it particularly promising for the development of new molecular quantum technologies, including quantum communication and quantum information processing, while also opening opportunities in polaritonic chemistry, such as controlling chemical reactivity, charge and electron transfer, and light-driven energy conversion at the molecular level.

### Limitations of the study

While the present results provide strong spectroscopic evidence for cavity-free JC-type coupling, field quantization, and few-molecule collective effects in the Ni_2_ system, the conclusions are based mainly on steady-state optical measurements together with analysis within the effective JC framework developed for this emitter-resonator integrated molecular system. In particular, the assignments of the *N* = 2 and *N* = 3 collective states and the $N\sqrt{N}\Omega_{1}$ scaling are inferred from spectral splittings and correlation-function simulations, while the detailed microscopic formation, configuration, and dynamics of the transient few-molecule ensembles in solution remain to be probed more directly. These limitations also point to clear opportunities for future work, including direct photon-correlation measurements at the Ni_2_ resonance; ultrafast time-resolved studies of the dressed-state dynamics; polarization-, concentration-, and temperature-dependent experiments on ensemble formation; and implementation of related chemically programmable M_2_ systems in immobilized or device-integrated platforms to test their potential for quantum photonics, quantum information science, and polaritonic chemistry.

## Resource availability

### Lead contact

Further information and requests for resources should be directed to and will be fulfilled by the lead contact, Chun Y. Liu (tcyliu@jnu.edu.cn).

### Materials availability

All unique reagents generated in this study are available from the lead contact upon reasonable request.

### Data and code availability

All data are available in the manuscript or the supplementary materials. The spectral raw data have been deposited at Mendeley (Mendeley Data: https://data.mendeley.com/datasets/gfck77w5jm) and are publicly available as of the date of publication. Additional information about the data will be made available from the corresponding author upon reasonable request.

## Acknowledgments

We thank Prof. Celso Jorge Villas-Boas and Dr. Ciro Micheletti Diniz for their helpful communications regarding the derivation of the collective coupling Hamiltonian. We acknowledge the primary financial support from the 10.13039/501100001809National Natural Science Foundation of China (grant nos. 22171107, 21971088, and 21371074), 10.13039/501100003453Natural Science Foundation of Guangdong Province (grant no. 2018A030313894), 10.13039/501100004024Jinan University, and the Fundamental Research Funds for the Central Universities.

## Author contributions

Conceptualization, C.Y.L.; methodology, M.M. and C.Y.L.; investigation, M.M., Y.N.T., Y.L.Z., Z.C.H., J.Z., G.Y.Z., and C.Y.L.; writing – original draft, C.Y.L. and M.M.; writing – review and editing, C.Y.L. and M.M.; funding acquisition, C.Y.L.; resources, C.Y.L.; supervision, C.Y.L.

## Declaration of interests

The authors declare no competing interests.

## STAR★Methods

### Key resources table

**Table:** 

REAGENT or RESOURCE	SOURCE	IDENTIFIER
**Chemicals, peptides, and recombinant proteins**		

Nickel(II) bromide	Aladdin	Cas# 13462-88-9
*n*-Butyllithium solution, 1.6 M in hexanes	Aladdin	Cas# 109-72-8
Triethyl orthoformate (99%)	Aladdin	CAS# 122-51-0
Tetrahydrofuran (Anhydrous, 99.9%)	Aladdin	CAS# 109-99-9
Ferrocenium hexafluorophosphate (97%)	Sigma Aldrich	CAS# 11077-24-0
Dichloromethane	Macklin	CAS# 75-09-2

### Method details

#### Synthesis of Ni_2_(DAniF)_4_

All manipulations were performed under an inert atmosphere using standard Schlenk techniques unless otherwise noted. The ligand HDAniF (0.9 g, 4 mmol) was dissolved in 20 mL of anhydrous THF in a 100 mL Schlenk flask. The solution was cooled to 0°C, and 2.5 mL of a 1.6 M solution of *n*-BuLi in hexane (4 mmol) was added dropwise with stirring. The mixture was allowed to warm to room temperature and stirred for 30 min to generate the lithium salt Li^+^(DAniF)^-^ *in situ*. To this solution, NiBr_2_ (0.22 g, 1 mmol) was added as a solid in one portion. The reaction mixture was heated to reflux (ca. 66°C) and stirred for 24 h. After cooling to room temperature, the resulting dark suspension was filtered through a frit to collect a microcrystalline black solid. The crude product was washed with 5 mL of cold THF and dried under vacuum. The black solid was then dissolved in a minimum amount of CH_2_Cl_2_ (ca. 5 mL), and the solution was layered with *n*-hexane (ca. 15 mL) to induce recrystallization. Dark crystalline product was collected by filtration, washed with *n*-hexane, and dried under vacuum to afford the product in ca. 40% yield (0.25 g).^17^^,^^18^

#### Spectroscopic measurements

All the spectroscopic measurements were conducted in dichloromethane (DCM) solution using quartz cell with light path length of 2 mm. The electronic (UV–Vis) spectra for the neutral and monocationic Ni_2_ complexes (5 × 10^−3^ mol L^−1^) were recorded on a Shimadzu UV-3600 (UV–VIS–NIR). The fluorescence spectra of the complexes were recorded on a Shimadzu RF-5301PC in DCM solution with a 5 nm slit width and a medium scan speed.

#### Simulation of the resonance fluorescence spectrum

The excitation (emitted at 418 nm) and emission (excited at 370 nm) spectra show a mirror relationship with respect to the resonance, with the first two pairs of peaks corresponding to the resonance spectra excited at 320–330 nm. Combination of these two spectra constructs a full resonance fluorescence that resolves all Rabi doublets for the single and collective coupling (*N* = 1–3) (Figure 3C). Figure S2 schematizes the simulated polaritonic transitions in the single and multiple photon processes. The Rabi splitting states for the *N* specified systems are given by R_*N*_ (±) = ω_0_ ± (*N*$\sqrt{N}$Ω_1_)/2 according to the collectively coupling scale (Equation 7), and consistently, the transition energies for the Mollow triplet sidebands are calculated from M_*N*_(±) = ω_0_ ± *N*Ω_1_′. Comparison of the simulated spectrum (Figure S2, dashed dark red trace) with the excitation-emission spectrum indicates that the R states are probed as spectral peaks (red lines), while the M states appear as the spectral valleys (blue lines). The calculated transition energies are in excellent agreement with the experimental observations, with the results strongly supporting the JC molecular system and the proposed collective coupling model. Some deviations for the Rabi doublets for *N* = 3 could be due to the intensified second sidebands at ω_0_ ± 2Ω_1_′ of the Mollow triplet at resonance (*N* = 2), which shifts the peak maxima toward each other. This peak vs. valley characteristics for the R and M states indicate that both the excitation and emission spectra are dominated by the vacuum Rabi splitting states for the single and collective coupling. It is notable that the excitation-emission spectrum shows two peaks at 320 nm and 473 nm (Figure S2, green lines with an upper arrow), which cannot be attributed to any R or M state. Interestingly, the transition energies for these two peaks can be scaled as ω_0_ ± (3Ω_1_′ ± Ω_1_/2). This indicates that they may emerge as coherent peaks as the R peaks are transformed into M peaks in the spectra by a π phase transition. This R to M state transformation is observed in the spectra for a dilute solution (Figure 6B). Indeed, the excitation spectrum exhibits the M_5_(+) peak at 300 nm, while the M_5_(−) is observed at 528 nm in the fluorescence spectrum excited at 450 nm (Figure S2), demonstrating the entanglement between these two states.

#### Analyses of the resonance fluorescence spectrum

For the systems differing in ensemble size (*N* = 1, 2 and 3), the polaritonic transition energies are extracted directly from the fluorescence spectra. The vacuum Rabi splittings (Ω_N_(exp)) are measured from the separations between the two peaks for corresponding Rabi doublets. The transition linewidths (FWHM) are determined from the simulated peak using a Lorentzian line profile in the selected spectrum, as show in Figures S1A–S1C. For the Rabi doublets, averaged linewidths Γ_aver_ are obtained by averaging the measured Γ(+) and Γ(−) for the upper and lower branches.

#### Detection of additional residues of the mollow triplet at resonance

While the spectra show three Rabi doublets for the single (*N* = 1) and collection (*N* = 2, 3) coupling to the scattering field, there appear more than three pairs of the Mollow triplet sidebands due to sideband excitation (Figures 6B and S2). These sidebands are characterized by the transition energies ω_0_ ± *N*Ω_1_′ (Ω_1_′ = 1450 cm^−1^). The M_3_(−) peak is coincident with the red satellite of the Mollow triplet at 430 nm, which can be attributed to excitation of the second red sideband of the Mollow triplet at resonance. The M_4_(−) peak at 490 nm is observed by excitation at 430 nm (Figure 6). In a different experimental entry, the spectra (Figure S4) exhibit the weak sideband at 525 nm when excited at 450 nm, which can be assigned to lowest-energy sideband M_5_(−) = ω_0_ − 5Ω_1_′ (528 nm). The appearance of the red M sidebands in the emission spectra with λ_ex_ ≥ 380 nm is attributed to sideband excitation. In contrast, the blue M sidebands cannot be resolved in the emission spectra because high energy excitation results in photon bunching at the resonance. However, in the excitation spectrum (Figure 3C) the absorption peaks at 300 nm and 288 nm correspond to M_5_(+) and M_6_(+), respectively.

#### Simulation of the second-order correlation function g^(2)^(τ)

##### Software and models

Origin 2021 software was used for the calculations and plotting of *g*^(2)^(τ). Time Domain Simulations were performed over τ = 0–50 fs. For each ensemble size (*N* = 1, 2, 3), the g^2^(τ) traces were plotted (Figures 5A–5C).

The CW formalism, i.e., Equation 6 in the main text^27^^,^^29^ was directly adopted as the computational models for the three systems (*N* = 1, 2 and 3) for *g*^(2)^(τ) simulations.

In Equation 6, γ is the bare radiative linewidth γ at resonance (Figure S1), and Ω = Ω(*N*)(exp) = 2g_*N*_ (≈πcΩ_*N*_) is set by the experimental splitting.

##### Conversion of linewidths to decay rates

Linewidths were converted to coherence times and decay rates as:

T_2_ = 1/(π*c*Γ_aver_), T_1_ = T_2_/2 (Γ_aver_ = γ_1_), with *c* = 2.998 × 10^10^ cm s^−1^. Angular frequencies were obtained with ω = 2π *c* Ω_*N*_(exp). For the CW simulation, γ = 1000 cm^−1^ was extracted from the central line at resonance.

##### Frequency-domain *g*^(2)^(τ) representations

The calculated time-domain g^(2)^(τ) traces for the three systems (*N* = 1, 2 and 3) derived based on the CW formalism were Fourier transformed into the Frequency-domain g^(2)^(τ) representations. The results are presented in Figure 5D.

### Quantification and statistical analysis

All spectral data processing and analyses were performed using Origin 2021 software (OriginLab Corporation). Spectral line positions and linewidths (full width at half maximum, FWHM) were determined by fitting the measured fluorescence and scattering spectra with Lorentzian or Gaussian profiles. The vacuum Rabi splittings (*Ω*_N(exp)_) were directly measured from the peak separations of the corresponding Rabi doublets, as shown in Figures 3C and S1. For each Rabi doublet, the average linewidth (*Γ*_aver_) was calculated by averaging the FWHMs of the upper and lower polariton branches. In this study, all reported spectral peak positions and splitting energies were obtained directly from the experimental spectra or from the fitted line profiles. No repeated measurements were performed to calculate standard deviations (SDs) or standard errors of the mean (SEM), as the analyses focused on the spectral line shapes and peak positions. All spectral fitting and simulations were performed using Origin 2021 software.

## References

[1] Haroche S., Raimond J.-M. (2006).

[2] Brune M., Schmidt-Kaler F., Maali A., Dreyer J., Hagley E., Raimond J.M., Haroche S. (1996). Quantum Rabi oscillation: A direct test of field quantization in a cavity. Phys. Rev. Lett..

[3] Houdré R., Stanley R.P., Ilegems M. (1996). Vacuum-field Rabi splitting in the presence of inhomogeneous broadening: Resolution of a homogeneous linewidth in an inhomogeneously broadened system. Phys. Rev. A.

[4] Jaynes E.T., Cummings F.W. (1963). Comparison of quantum and semiclassical radiation theories with application to the beam maser. Proc. IEEE.

[5] Shore B.W., Knight P.L. (1993). The Jaynes-Cummings Model. J. Mod. Opt..

[6] Thompson R.J., Rempe G., Kimble H.J. (1992). Observation of Normal-Mode Splitting for an Atom in an Optical Cavity. Phys. Rev. Lett..

[7] Fink J.M., Göppl M., Baur M., Bianchetti R., Leek P.J., Blais A., Wallraff A. (2008). Climbing the Jaynes–Cummings ladder and observing its nonlinearity in a cavity QED system. Nature.

[8] Chikkaraddy R., de Nijs B., Benz F., Barrow S.J., Scherman O.A., Rosta E., Demetriadou A., Fox P., Hess O., Baumberg J.J. (2016). Single-molecule strong coupling at room temperature in plasmonic nanocavities. Nature.

[9] Esteban R., Borisov A.G., Nordlander P., Aizpurua J. (2012). Bridging quantum and classical plasmonics with a quantum-corrected model. Nat. Commun..

[10] Savage K.J., Hawkeye M.M., Esteban R., Borisov A.G., Aizpurua J., Baumberg J.J. (2012). Revealing the quantum regime in tunnelling plasmonics. Nature.

[11] Benz F., Schmidt M.K., Dreismann A., Chikkaraddy R., Zhang Y., Demetriadou A., Carnegie C., Ohadi H., de Nijs B., Esteban R. (2016). Single-molecule optomechanics in “picocavities”. Science.

[12] Tavis M., Cummings F.W. (1968). Exact solution for an N-molecule-radiation-field Hamiltonian. Phys. Rev..

[13] Qin W., Kockum A.F., Muñoz C.S., Miranowicz A., Nori F. (2024). Quantum amplification and simulation of strong and ultrastrong coupling of light and matter. Phys. Rep..

[14] Frisk Kockum A., Miranowicz A., De Liberato S., Savasta S., Nori F. (2019). Ultrastrong coupling between light and matter. Nat. Rev. Phys..

[15] Dicke R.H. (1954). Coherence in Spontaneous Radiation Process. Phys. Rev..

[16] Tan Y.N., Meng M., He Z.C., Cheng T., Luo J.B., Wang X.D., Zhong Z.H., Zhou J., Zhu G.Y., Xiao X. (2023). Zero-dimensional molecular exciton-polaritons in cavity-free solutions. Cell Rep. Phys. Sci..

[17] Cotton F.A., Matusz M., Poli R., Feng X. (1988). Dinuclear formamidinato complexes of nickel and palladium. J. Am. Chem. Soc..

[18] Berry J.F., Bothe E., Cotton F.A., Ibragimov S.A., Murillo C.A., Villagrán D., Wang X. (2006). Metal–Metal Bonding in Mixed Valence Ni_2_^5+^ Complexes and Spectroscopic Evidence for a Ni_2_^6+^ Species. Inorg. Chem..

[19] WebElements Atomic sizes of elements. https://webelements.com/rhenium/atom_sizes.html.

[20] Mollow B.R. (1969). Power spectrum of light scattered by two-level systems. Phys. Rev..

[21] López Carreño J.C., del Valle E., Laussy F.P. (2017). Photon correlations from the Mollow triplet. Laser Photon. Rev..

[22] Wrigge G., Gerhardt I., Hwang J., Zumofen G., Sandoghdar V. (2008). Efficient coupling of photons to a single molecule and the observation of its resonance fluorescence. Nat. Phys..

[23] Villas-Boas C.J., Máximo C.E., Paulino P.J., Bachelard R.P., Rempe G. (2025). Bright and Dark States of Light: The Quantum Origin of Classical Interference. Phys. Rev. Lett..

[24] Villas-Boas C.J., Diniz C.M. (2025). Dark States of Light and the Hidden Energy in Thermal Radiation Detection. arXiv.

[25] Meng M., Tan Y.N., He Z.C., Zhong Z.H., Zhou J., Zhou Y.L., Zhu G.Y., Liu C.Y. (2026). Quadruply Bonded Mo_2_ Molecules: An Innate Emitter-Resonator Quantum System in Free Space. arXiv.

[26] Walls D.F. (1983). Squeezed states of light. Nature.

[27] Carmichael H. (1991).

[28] Carmichael H.J., Walls D.F. (1976). Proposal for the measurement of the resonant Stark effect by photon correlation techniques. J. Phys. B Atom. Mol. Phys..

[29] Mollow B.R. (1972). Absorption and emission line-shape functions for driven atoms. Phys. Rev. A..

[30] Carmichael H.J., Walls D.F. (1976). A quantum-mechanical master-equation treatment of the dynamical Stark effect. J. Phys. B: At. Mol. Phys.

[31] Kimble H.J., Dagenais M., Mandel L. (1977). Photon Antibunching in Resonance Fluorescence. Phys. Rev. Lett..

[32] Basché T., Moerner W.E., Orrit M., Talon H. (1992). Photon Antibunching in the Fluorescence of a Single Dye Molecule Trapped in a Solid. Phys. Rev. Lett..

[33] Lounis B., Moerner W.E. (2000). Single photons on demand from a single molecule at room temperature. Nature.

[34] Foster G.T., Mielke S.L., Orozco L.A. (2000). Intensity correlations in cavity QED. Phys. Rev. A.

[35] Meystre P., Zubairy M.S. (1982). Squeezed states in the Jaynes-Cummings model. Phys. Lett..

[36] Carmichael H.J., Lane A.S., Walls D.F. (1987). Resonance Fluorescence from an Atom in a Squeezed Vacuum. Phys. Rev. Lett..

[37] Griffin R.D., Harris S.M. (1982). Two-atom resonance fluorescence including the dipole-dipole interaction. Phys. Rev. A.

[38] Darsheshdar E., Hugbart M., Bachelard R., Villas-Boas C.J. (2021). Photon-photon correlations from a pair of strongly coupled two-level emitters. Phys. Rev. A.

[39] Shen F., Gao J., Senin A.A., Zhu C.J., Allen J.R., Lu Z.H., Xiao Y., Eden J.G. (2007). Many-body dipole-dipole interactions between excited Rb atoms probed by wave packets and parametric four-wave mixing. Phys. Rev. Lett..

[40] López Carreño J.C., Sánchez Muñoz C., Sanvitto D., Del Valle E., Laussy F.P. (2015). Exciting polaritons with quantum light. Phys. Rev. Lett..

[41] Beige A., Hegerfeldt G.C. (1998). Transition from antibunching to bunching for two dipole-interacting atoms. Phys. Rev. A.

[42] Pucci L., Roy A., Santo T.S.d.E., Kaiser R., Kastner M., Bachelard R. (2017). Quantum effects in the cooperative scattering of light by atomic clouds. Phys. Rev. A.

[43] Palma G.M., Knight P.L. (1989). Phase-sensitive population decay: The two-atom Dicke model in a broadband squeezed vacuum. Phys. Rev. A.

