# Supplementary material for: Strong light-matter coupling and field quantization in a dinickel molecular resonator
Source: iScience. 2026 Aug 6;29(8):117076. doi: 10.1016/j.isci.2026.117076 (PMC13475667; doi:10.1016/j.isci.2026.117076)
Supplement: Supplementary file 1 [file mmc1.pdf]

iScience, Volume *29*

Supplemental information

Strong light-matter coupling and field quantization in a dinickel molecular resonator

Miao Meng, Ying Ning Tan, Yu Li Zhou, Zi Cong He, Zi Hao Zhong, Jia Zhou, Guang Yuan Zhu, and Chun Y. Liu

Supplementary Materials for

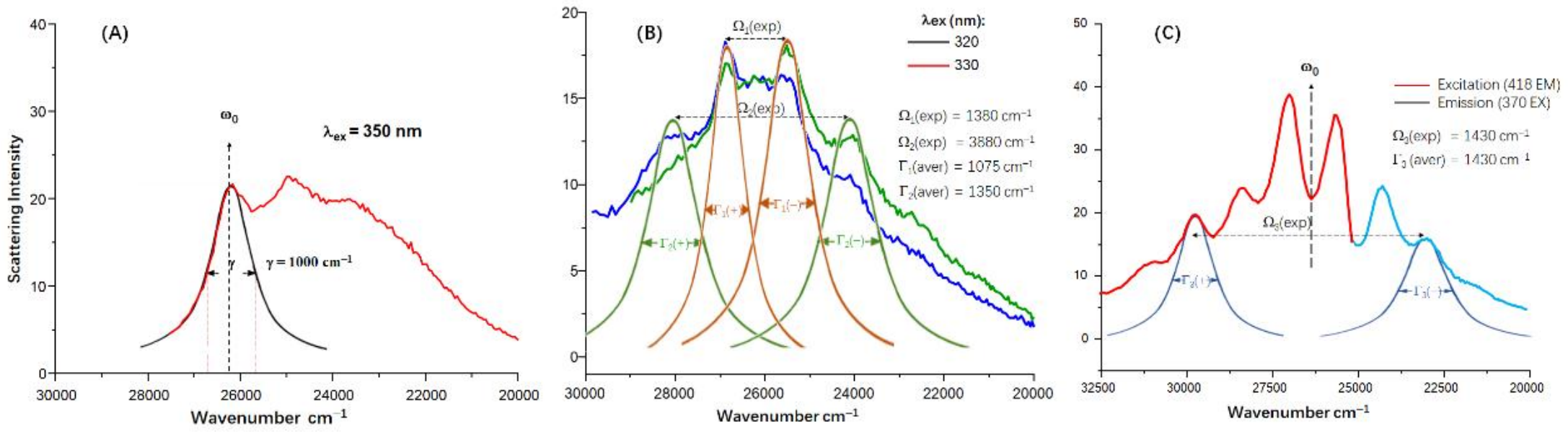

Figure S1. Determinations of the Rabi splittings (cm^{-1}) and linewidths (FWHM) from the resonance fluorescence spectra. (A) Deconvolution of the resonance peak at 380 nm in the fluorescence spectrum excited at 350 nm using a Lorentzian line profile. The linewidth (FWHM) γ = 1000 cm^{-1}. (B) Determination of the transition energies (cm^{-1}), linewidth Γ (FWHM) (cm^{-1}) and the Rabi splitting (Ω(exp)) of the sidebands for N = 1 and 2. (C) Deconvolution of the sidebands in the fluorescence spectrum combined with the excitation (emission at 418 nm (23923 cm^{-1})) and the emission (excitation at 370nm (27027 cm^{-1})) spectra using a Lorentzian line profile to determine coupling strength Ω_3(exp) and the linewidth (FWHW) Γ for $N = 3$.

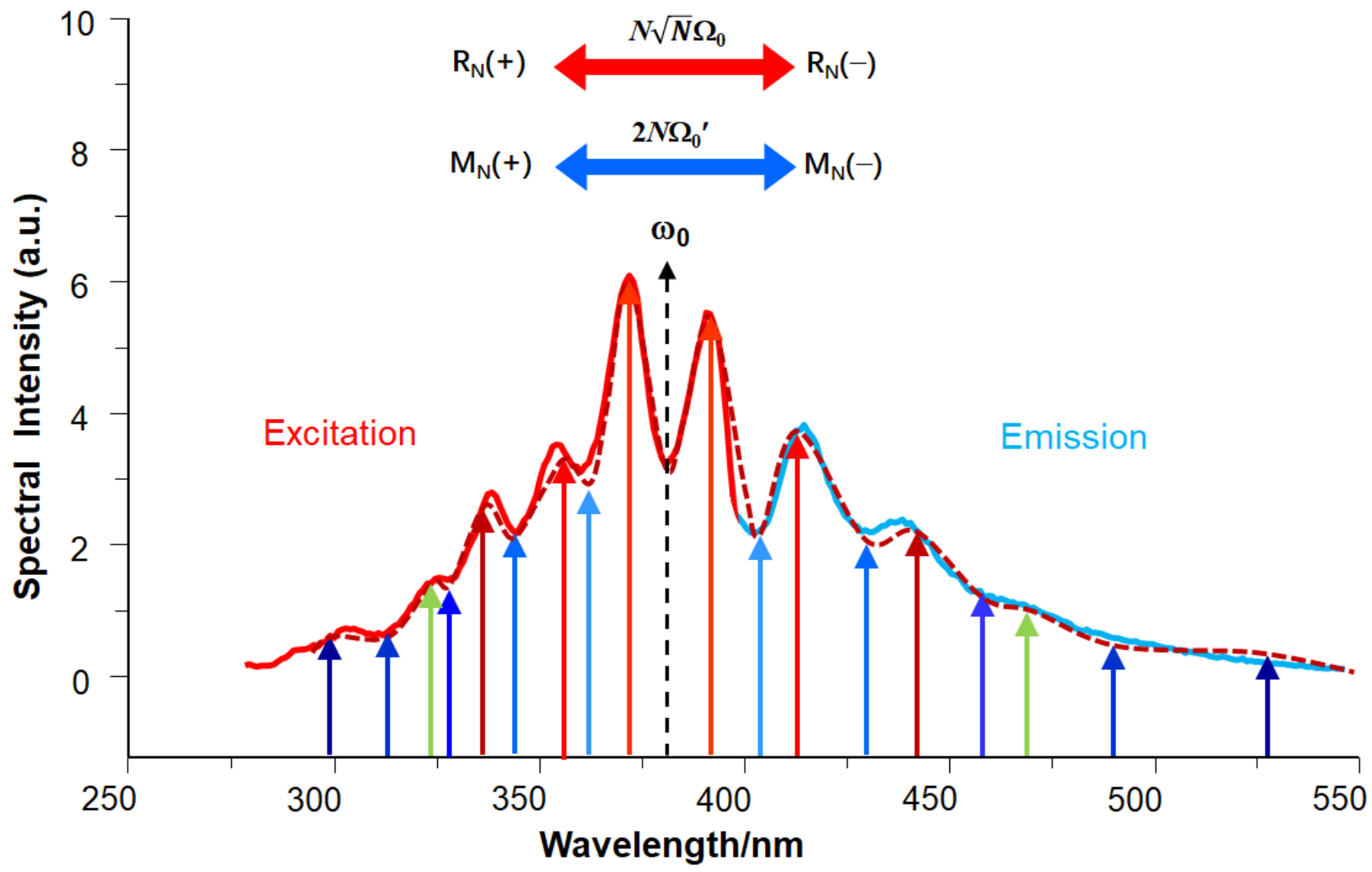

Figure S2. Simulations of the polaritonic transitions based on the collective coupling scale for the JC molecule system. The transition energies for the Rabi doublets (R) and the Mollow triplet sidebands (M) are calculated from $R_N(\pm) = \omega_0 \pm (N\sqrt{N}\Omega_1)/2$ and $M_N(\pm) = \omega_0 \pm N\Omega_1'$, respectively.

The R and the M transitions are indicted by the red and blue vertical lines with a upper arrow, respectively. Two coherent peaks at 320 nm and 473 nm (upper-arrowed green lines) are characterized by $\omega_0 \pm (3\Omega_1' \pm \Omega_1/2)$. For each color series, color changes from pale to dark correspond to N increases from 1 to 3 for the R peaks and to 5 for the M peaks. The simulated spectrum is generated by smoothly tracing the R and M transitions with the intensity similar to those in the experimental spectra.

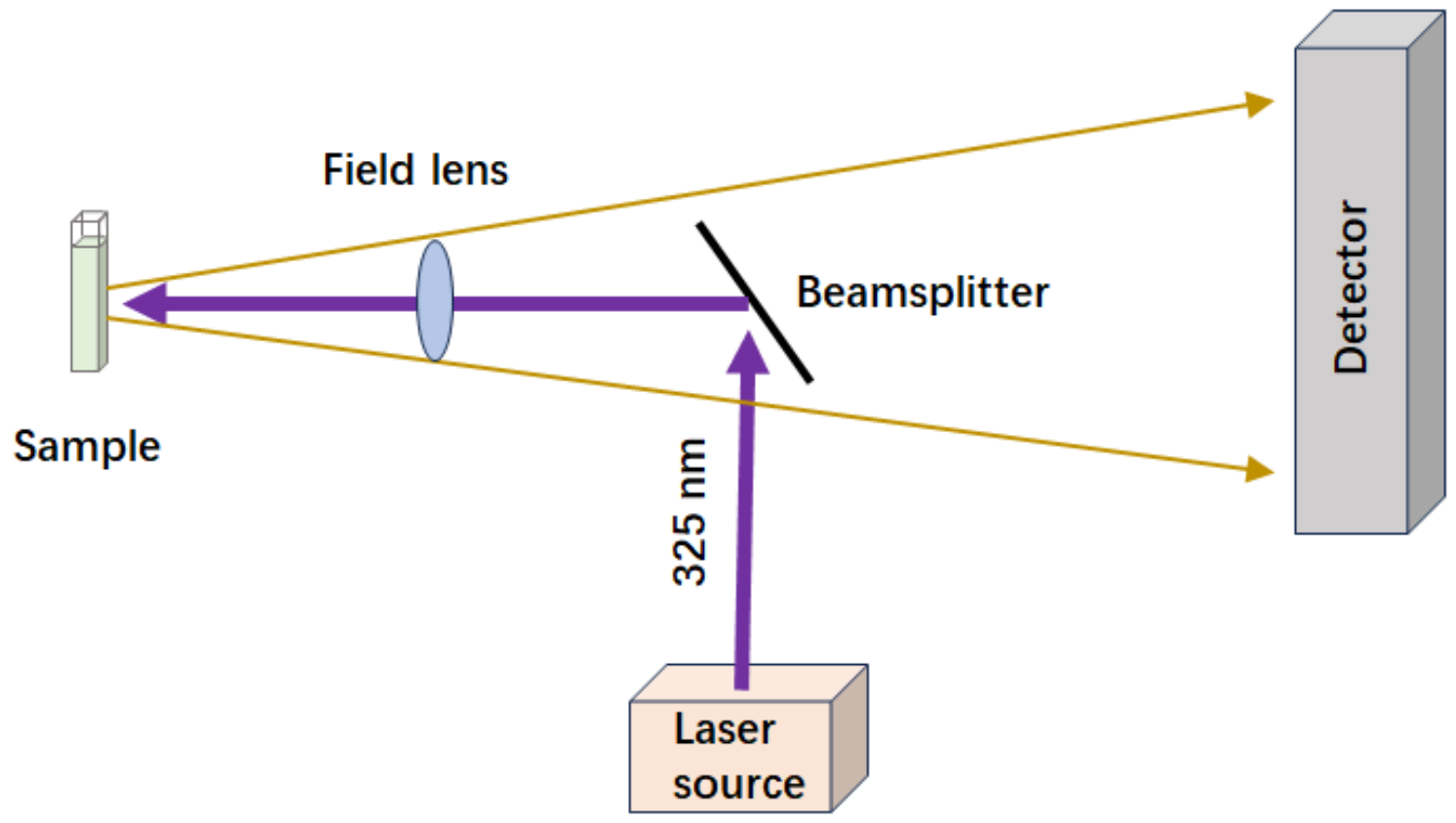

Figure S3. Schematical illustration of experimental setups for recording the scattering spectra of the Ni_2 complex. Figure S3 shows the schematic of the experimental setup for recording the polariton parametric scattering of $Ni_2(DAniF)_4$. Scattering spectra of the Ni_2 complex were recorded for the DCM solutions in a quartz cell with a light path length of 2 mm. The complex samples were pumped with a 325 nm continuous-wave pulsed laser. The spectra were analyzed using a spectrometer (Princeton Instruments SP2500, Acton) equipped with a silicon charge-coupled device. Multiple measurements with continuously varying concentrations and laser intensities were carried out to study the concentration and power dependencies. Solvent (complex blank) supectrum was recorded at the same conditions.

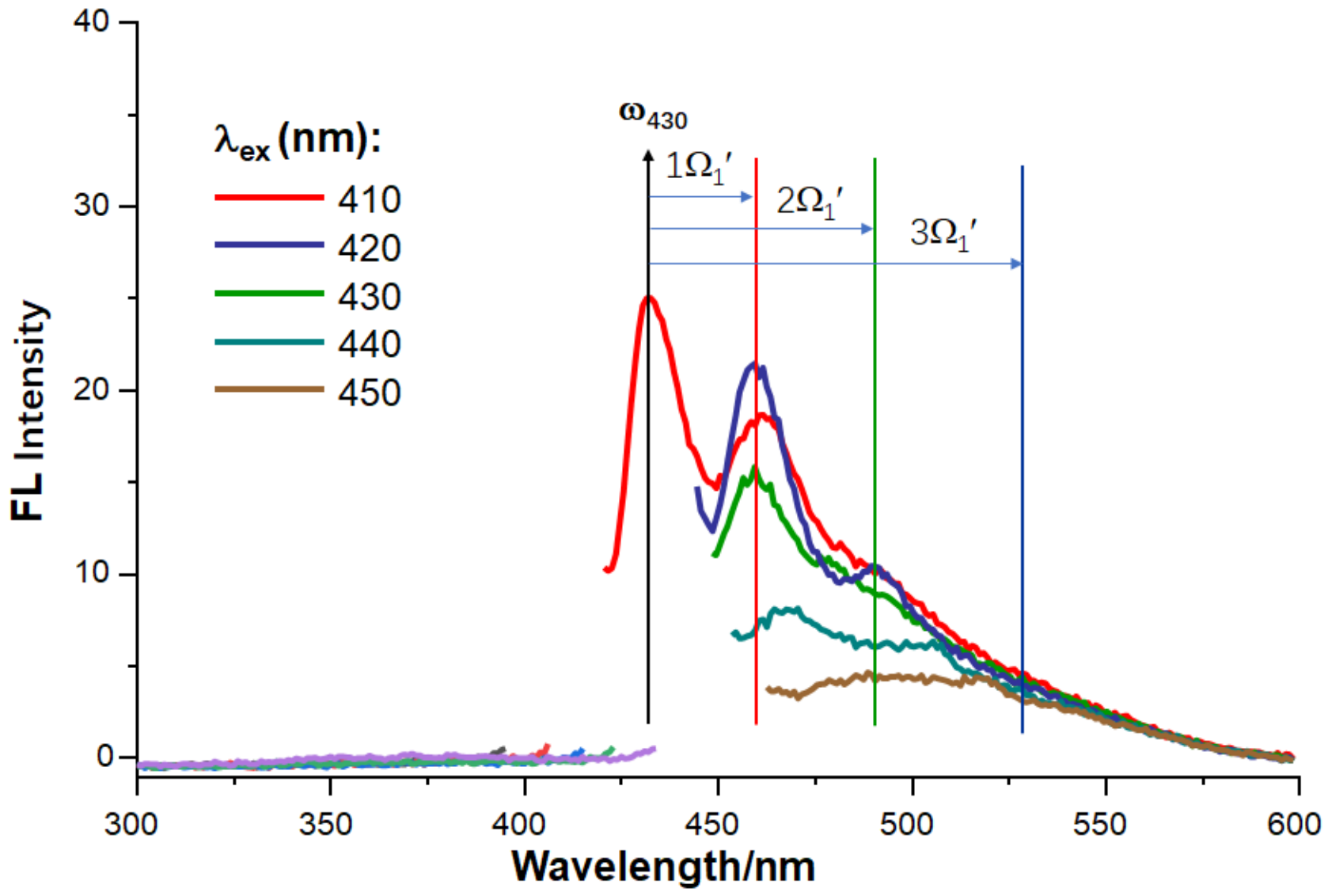

Figure S4. Observation of additional Mollow triplet sidebands in the fluorescence spectra. The measurements allow observation of two additional M sidebands. The intervals between peaks remain 1450 cm^{-1} (Ω_1'). The ($\omega_0 - 4\Omega_1'$) peak is observed at (490 nm by excitation at 420 nm, as expected. The lowest energy M peak ($\omega_{430} - 3\Omega_1'$) or ($\omega_0 - 5\Omega_1'$) appears at 525 nm ($\lambda_{ex}$ = 450 nm), deviated from the predicted position at 528 nm. This experimental error may be due to the limit of the spectral window of the complex.
